# Determining Proportion of Exfoliative Vaginal Cell during Various Stages of Estrus Cycle Using Vaginal Cytology Techniques in Aceh Cattle

**DOI:** 10.1155/2016/3976125

**Published:** 2016-02-10

**Authors:** Tongku N. Siregar, Juli Melia, Rohaya, Cut Nila Thasmi, Dian Masyitha, Sri Wahyuni, Juliana Rosa, Nurhafni, Budianto Panjaitan, Herrialfian

**Affiliations:** Faculty of Veterinary Medicine, Syiah Kuala University, Banda Aceh 23111, Indonesia

## Abstract

The aim of this study was to investigate the period of estrus cycle in aceh cattle, Indonesia, based on vaginal cytology techniques. Four healthy females of aceh cattle with average weight of 250–300 kg, age of 5–7 years, and body condition score of 3-4 were used. All cattle were subjected to ultrasonography analysis for the occurrence of corpus luteum before being synchronized using intramuscular injections of PGF2 alpha 25 mg. A vaginal swab was collected from aceh cattle, stained with Giemsa 10%, and observed microscopically. Period of estrus cycle was predicted from day 1 to day 24 after estrus synchronization was confirmed using ultrasonography analysis at the same day. The result showed that parabasal, intermediary, and superficial epithelium were found in the vaginal swabs collected from proestrus, metestrus, and diestrus aceh cattle. Proportions of these cells in the particular period of estrus cycle were 36.22, 32.62, and 31.16 (proestrus); 21.33, 32.58, and 46.09 (estrus); 40.75, 37.58, and 21.67 (metestrus); and 41.07, 37.38, and 21.67 (diestrus), respectively. In conclusion, dominant proportion of superficial cell that occurred in estrus period might be used as the base for determining optimal time for insemination.

## 1. Introduction

The changes during normal estrus cycle relate to the basic concept of the ovulation process, regression of the corpus luteum, pregnancy, and birth. The ovulation process is related to estrus and mating. A good estrus detection will be able to determine the optimum time for insemination [1]. However, the accuracy to detect estrus to determine the optimum time for insemination is below 50% [2].

There are many methods to identify the estrus cycle and the optimal determination mating time; they are estrus observation [3], measurement of steroid level, vaginal cytology [4], ultrasound, and rectal palpation. In aceh cattle, the estrus observation for optimal mating time determination is limited by the low performance of estrus [5] especially in the event of environmental heat stress [6] whereas the steroid examination is relatively noneconomical and has a longer execution time. The vaginal cytology is a simple technique and alternative that can be used by practitioners to characterize the reproduction cycle of the estrus [7]. The research on female collared peccary showed that vaginal cytology can be used as an estrus cycle predictor [8].

The research in the vaginal cytology method using animals has been done for many times, for example, in a dog [9], cow [10, 11], goat [12], and deer [13]. The examination of the vaginal cytology during estrus cycle has been conducted for clinically appearing estrus symptoms [14] and steroid concentration [4, 12]. However, there are many chances of making a mistake in determining the estrus cycle based on the observation of the clinical symptoms compared to determining the estrus cycle based on the ultrasound observation (USG), whereas the steroid examination is relatively noneconomical and has more execution time. In aceh cattle, the determination of the estrus cycle based on vaginal cytology imaging has not been reported.

## 2. Material and Methods

This research uses four adult females of aceh cattle; they were not in a pregnant condition, clinically healthy, and aging from 5 to 7 years with a weight of 250–300 kg. The cattle had a good body condition score with good criteria between 3 and 4 on a score scale of 5. Additionally, all cattle have normal reproductive organs marked by showing at least twice regular estrous cycle, ever pregnant and having birth, and also free from endometritis and pyometra.

### 2.1. Estrus Synchronization

In order to get a day 0 estrus cycle (estrus period, standing heat), all cattle had estrus synchronization using luteolytic dosage PGF2*α* injections (25 mg, Lutalyse*™*, Pharmacia & Upjohn Company, Pfizer Inc.). The injections were done twice with interval of 11 days. Twenty-four hours after the last PGF2*α* injections, examination using USG was performed every day during the estrus cycle.

### 2.2. The Vaginal Cytology Preparation

The vaginal cytology preparation was done according to the instructions of Ola et al. [15]. Observations were done using light microscope with objective lens magnification of 40 × 100. The observed vaginal epithelial cells (superficial, intermediate, and parabasal) were counted according to each group to the determined phases of the estrus cycle based on the USG observation results.

### 2.3. USG Examinations

The cattle were placed in a pinned cage and the USG device (MINDRAY DP3300VET, Shenzhen Mindray Bio-Medical Electronic Co., Ltd., China) was placed on a safe place away from the cattle and easy to be operated by the operator. Feces were released from the cattle's rectum; then, a manual exploration was done from the topography of the cattle's reproduction tracts before the USG examination. Ovaries were located on the underside of the left and right uterine cornua. Diameter CL and follicles in the ovaries were measured using internal caliper on ultrasound, that is, the distance between the two points of the longest axis by axis with units cm. Furthermore, imaging of vagina, cervix, and uterus body was captured in long axis of craniocaudal view of pubic region. When the transducer was moved to the lateral, the cornua uterus would be seen in a cross section state.

### 2.4. Measurement of Serum Progesterone and Estradiol

Collection of blood (10 mL) for examination of progesterone and estradiol concentrations conducted during the estrus cycle begins on day 0 (time of estrus) and ends at the next estrus. Serum was recovered by centrifugation (15 minutes at 2,500 rpm) and stored at −20°C until being assayed for serum progesterone and estradiol concentrations using commercial progesterone and estradiol ELISA kit (DRG Instruments GmbH, Germany).

### 2.5. Data Analysis

Vaginal cells percentages among the stages of estrus cycle were compared by Chi-square test. The mean progesterone and estradiol concentrations between groups were analyzed by one-way ANOVA.

## 3. Results and Discussion

Several types of cells were observed in the mucosal surface of vagina during estrous phases. Those cells were parabasal, intermediate, and superficial cells. This observation was according to the reports of Najamudin et al. [13]. The vaginal epithelial cell consists of three types and they were the parabasal, intermediate, and superficial cell. The parabasal cell was the small epithelial cell found in the vagina, having a round shape, and nucleus was bigger than the cytoplasm. The intermediate cell has variation of shapes and has size 2-3 times compared to the parabasal cell. The superficial cell was the large sized cells and had a polygonal and flat shape but did not always have the pyknotic nucleus. The proportion of each epithelial cell during the estrus cycle is shown in Table 1.

Based on the standard and characteristic of epithelial cell in proestrus and estrus phase, the proportion of parabasal, intermediate, and superficial cell was found with measurement of 36.22, 32.62, and 31.16 and 21.33, 32.58, and 46.09, respectively. The collected proportion from this research was according to Widiyono et al. [12], in which the largest epithelial cell proportion in the estrus phase is the superficial cells or superficial and intermediate cells in proestrus and estrus phase. The results showed that the proportion of the superficial cells was higher during proestrus (31.16) and increased during estrus (46.09) and showed significant differences with metestrus and diestrus phase (*P* < 0.05). The proportion of superficial cells during metestrus and diestrus was, respectively, 21.67 and 21.54 (*P* > 0.05).

Even though the population of the superficial cells is dominant in this phase, it is still relatively low in contrast to the superficial cell in estrus of dogs that reached 90% [9].

In estrus phase, estrogen hormone will increase activeness in uterus wall; it causes hypersecretion in epithelial cells of the uterus and vagina, so that the superficial cells followed the vaginal peel. In this research, the estradiol concentrations in proestrus, estrus, metestrus, and diestrus phase were 171.99 + 11.30, 223.13 + 9.50, 10.05 + 98.03, and 67.37 + 8.75 pg mL^−1^, respectively. Increasing concentrations of estradiol in proestrus and estrus phase may be related to the high proportion of superficial cells. In bligon goat, the percentage of superficial cell was found to be 32.25% when estradiol was on level 247.77 pg dL^−1^, 25.50% when the uterus and estradiol level was 246.17 pg dL^−1^, and lowered to level of 12.22% when the lowest estradiol level was at 211.25 pg dL^−1^. The average concentration of progesterone was different on the stage of aceh cattle cycle. At the time of proestrus (2-3 days), progesterone level was 0.97 + 0.21 ng mL^−1^. The concentration reached its lowest level during estrus (1-2 days), that is, 0.12 + 0.02 ng mL^−1^. Metestrus and diestrus phase length were 13–16 days with average concentration of progesterone being 0.10 ± 1.67 ng mL^−1^. When progesterone becomes dominant in metestrus and diestrus phase, the number of larger cells was sharply reduced [16], so that the epithelial cells were dominated by parabasal cells. In this study, the proportion of parabasal cells in metestrus and diestrus phase was, respectively, 40.75 and 41.07 and significantly (*P* < 0.05) higher in comparison with proestrus and diestrus phase. From the USG confirmation results, it could be seen that the estrus phase was marked by outbreak of the dominant follicle or ovulation. This indicated that the determination of the cycle phase in this research was correctly located in the estrus phase. The estrus phase in aceh cattle was 1-2 days. Different from the obtained results by Mingoas and Ngayam [11] with zebu cattle, the estrus phase in this research did not account for the cells undergoing cornification. The superficial epithelial cell which is not nucleus often undergoes cornification or keratinization which serves to protect the vaginal mucosa from irritation at the time of copulation. The loss of the epithelial cell nucleus in the estrus phase can be caused by the keratinization process. The keratinized cells were found individually separated from the other cells. Degeneration of those cells is caused by keratin substance blocking nutrient diffusion from the capillaries in the bondage tissue. In this phase, desquamation of superficial epithelial cells was also detected.

In the diestrus phase, population of superficial cells decreased and happened vice versa on the increase of intermediate and parabasal cells. Towards the end of diestrus, degeneration was caused by shaping vacuole on cytoplasm and nucleus was cornered to the side. In metestrus and diestrus phase, the proportions of parabasal, intermediate, and superficial cells each were 40.75, 37.58, and 21.67 and 41.07, 37.38, and 21.6, respectively. These proportions were according to Solis et al. [17] that parabasal cells were very dominant in phase before estrus in sheep, followed by intermediate and superficial cells. Parabasal cells (epithelial cells with huge nucleus) were the smallest of the epithelial cell type in vaginal peel. Parabasal cells are usually found on the estrus and anestrus phase. The observation results showed that the intermediate cells dominated during the estrus cycle. The same phenomenon was also reported by Widiyono et al. [12] in bligon goats. The results showed that the proportion of intermediate cells dominates the cell vaginal swabs during the estrus cycle. The same phenomenon was also reported by Widiyono et al. [12] in bligon goats. The proportions of intermediate cells were similar (*P* > 0.05) during proestrus (32.62), estrus (32.58), metestrus (37.58), and diestrus (37.38) obtained in this research.

## 4. Conclusion

The Indonesia aceh cattle vaginal epithelial cells during the estrus cycle consist of parabasal, intermediate, and superficial cell. In estrus phase, the superficial epithelial cell is the most dominant in comparison to the metestrus, diestrus, or even proestrus phase.

## Figures and Tables

**Table 1:** Characteristic and proportion of the Indonesian aceh cattle vaginal epithelial cell during the estrus cycle.

Phase	Duration period Estrus (days)	Vaginal epithelial cell proportion (%)			USG imaging	Steroid concentration	
		Parabasal	Intermediate	Superficial		Estrogen	Progesterone (ng/mL)
Proestrus	2-3	36.22^a,A^	32.62^a,A^	31.16^a,A^	Dominant follicle with a diameter of 10.60 ± 1.63 mm; CL starts to be lysis, and uterus is not yet enlarged		0.97 ± 0.21^a^

Estrus	1-2	21.33^b,A^	32.58^a,B^	46.09^b,C^	Dominant follicle (3rd DF) sized 13.75 + 1.71 mm; CL starts to be lysis; enlargement of the corpus uterus, a decrease in endometrial thickness		0.12 ± 0.02^a^

Metestrus	3-4	40.75^c,A^	37.58^a,A^	21.67^c,B^	Dominant follicle (1st DF) sized 10.00 ± 1.83 mm; CL present; a decrease in diameter of the uterus		1.40 ± 0.0^b^

Diestrus	10–12	41.07^c,A^	37.38^a,A^	21.54^c,B^	Diameter of the dominant follicle (2nd DF) with a size up to 8.75 ± 0.50 mm; CL present in the fixed/functional phase; stabilized uterus		1.94 + 0.1^b^

^a,b,c^The different superscript in the same column indicates significant differences (*P* < 0.05).

^A,B,C^The different superscript in the same line indicates significant differences (*P* < 0.05).
